# Enhanced histamine H2 excitation of striatal cholinergic interneurons in L-DOPA-induced dyskinesia

**DOI:** 10.1016/j.nbd.2015.01.003

**Published:** 2015-02-04

**Authors:** Sean Austin O. Lim, Rong Xia, Yunmin Ding, Lisa Won, William J. Ray, Stephen A. Hitchcock, Daniel S. McGehee, Un Jung Kang

**Affiliations:** 1 Committee on Neurobiology, University of Chicago, Chicago, IL 60637; 2 Department of Neurology, University of Chicago, Chicago, IL 60637; 3 Department of Anesthesia & Critical Care, University of Chicago, Chicago, IL 60637; 4 Department of Neurology, Columbia University, New York, NY 10032; Envoy Therapeutics, Inc, Jupiter, Florida 33458

**Keywords:** 6-OHDA lesion, Aphakia, Cholinergic interneuron, Famotidine, Histamine, L-DOPA induced dyskinesia, Parkinson's disease, Striatum

## Abstract

Levodopa is the most effective therapy for the motor deficits of Parkinson's disease (PD), but long term treatment leads to the development of L-DOPA-induced dyskinesia (LID). Our previous studies indicate enhanced excitability of striatal cholinergic interneurons (ChIs) in mice expressing LID and reduction of LID when ChIs are selectively ablated. Recent gene expression analysis indicates that stimulatory H2 histamine receptors are prefentially expressed on ChIs at high levels in the striatum, and we tested whether a change in H2 receptor function might contribute to the elevated excitability in LID. Using two different mouse models of PD (6-hydroxydopamine lesion and *Pitx3^ak/ak^* mutation), we chronically treated the animals with either vehicle or L-DOPA to induce dyskinesia. Electrophysiological recordings indicate that histamine H2 receptor-mediated excitation of striatal ChIs is enhanced in mice expressing LID. Additionally, H2 receptor blockade by systemic administration of famotidine decreases behavioral LID expression in dyskinetic animals. These findings suggest that ChIs undergo a pathological change in LID with respect to histaminergic neurotransmission. The hypercholinergic striatum associated with LID may be dampened by inhibition of H2 histaminergic neurotransmission. This study also provides a proof of principle of utilizing selective gene expression data for cell-type-specific modulation of neuronal activity.

## Introduction

The majority of motor symptoms in Parkinson's disease (PD), including bradykinesia, rigidity, and tremor are most effectively remedied by 3,4-dihydroxy-L-phenylalanine (L-DOPA), the biochemical precursor to dopamine. An unfortunate consequence of long term L-DOPA treatment is that within 4-6 years, more than half of patients develop L-DOPA-induced dyskinesia (LID), which is characterized by potentially debilitating uncontrolled, hyperkinetic movements (Hauser et al., 2007; Jenner, 2008). At present, a non-competitive antagonist of N-methyl-d-aspartate (NMDA) glutamate receptors, amantadine, which also has anticholinergic effect, is the only drug shown to alleviate LID clinically (Fernandez, 2012). However, pharmacotherapy with amantadine has limited efficacy and potential adverse side effects (Thomas et al., 2004; Wolf et al., 2010). Therefore, new approaches to LID therapy are needed to alleviate LID symptoms without interfering with the anti-parkinsonian effect of L-DOPA.

Unilateral 6-OHDA lesioned rodents are the most widely used animal models for PD and LID (Lundblad et al., 2004; Lundblad et al., 2005; Pavon et al., 2006; Santini et al., 2009). While important aspects of these conditions are present in these animals, this approach requires intra-cranial infusion of the neurotoxin 6-OHDA, leading to variable degrees of striatal DA denervation. These animals require extensive post-operative care and the dose of L-DOPA used to induce behavioral responses varies widely (1 mg/kg up to 25 mg/kg), depending on the severity of striatal DA denervation. In contract, Pitx3^ak/ak^ mice display the selective loss of nigral dopamine neurons from birth due to a naturally occurring mutation in the Pitx3 gene (Hwang, et al., 2003; Nunes et al., 2003). This is similar to mild PD with early onset of the disease. These animals display bilateral striatal DA denervation and L-DOPA treatment affects behavior on both sides of the body, while the unilateral 6-OHDA lesion affects only the contralateral side. Thus, the bilateral vs. unilateral response to L-DOPA results in the significant phenotypic difference in LID when treated with repetitive L-DOPA. In Pitx3^ak/ak^ , LID engages both sides of the body, inducing unique abnormal involuntary limb movements with the body in a vertical position, while in 6-OHDA lesioned rodents, only one side of the body displays LID (Ding et al, 2007; (Solis et al., 2014). The LID phenotype in Pitx3^ak/ak^ is similar to that in adult wild type mice treated with MPTP and a high dose of L-DOPA in both biochemical and behavioral level (Nicholas, 2007) as well as other genetic models of bilateral dopamine depletion (Chartoff et al., 2001; Kim et al., 2000). While these animal models have advantages and limitations, we have utilized both to optimally assess the potential relevance of our observations to human PD patients.

Abnormal striatal cholinergic tone contributes to LID (Ding et al., 2011). In the dorsal striatum, acetylcholine is synthesized and released by a class of interneurons that are believed to be analogs of the ‘tonically active neurons’ (TANs) described in primates, which are involved in associative learning (Aosaki et al., 1995, 1994; Kimura et al., 1984). In the mouse, these cholinergic interneurons (ChIs) comprise between 1-2% of all striatal cells, but through their extensive arborization, they affect the activity of both dopaminergic nerve terminals and the medium spiny neurons that encode striatal output (Bolam et al., 1984; Calabresi et al., 2000; Threlfell et al., 2012; Wilson et al., 1990). Dysfunction in striatal cholinergic tone has been observed in movement disorders including dystonia and Huntington's disease (Farrar et al., 2011; Sciamanna et al., 2012), and our previous research implicates a role for a change in ChI physiology in LID (Ding et al., 2011; Won et al., 2014). In extracellular recordings of ChI firing rate, enhanced baseline excitability and hypersensitivity to dopamine are both associated with LID in mouse models of PD. Behavioral expression of LID is also associated with an increase in phosphorylated extracellular signal-regulated kinase levels in ChIs. Both the altered ChI physiology and behavioral expression of LID are dependent on the mitogen-activated protein kinase signaling transduction pathway (Ding et al., 2011). Furthermore, ablation of striatal ChIs reduces LID (Won et al., 2014). These data indicate that modulation of striatal cholinergic tone may be a potential target for LID therapy.

Inhibition of muscarinic receptors with the M1-selective antagonist, dicyclomine (Giachetti, et al., 1986) can reduce the severity of LID (Ding et al., 2011). As a therapy, blocking M1 receptors is prone to side effects as these receptors are widely expressed in the brain and affect a number of networks and physiological processes (Smythies, 2005). The histaminergic system may be an alternative target in LID therapy, as changes in histamine signaling have been implicated in the pathophysiology of PD and LID. In postmortem brain samples from patients with PD or rodent PD models, extracellular histamine levels and the density of histaminergic fibers are increased in several brain regions, including the basal ganglia and substantia nigra pars compacta (Anichtchik et al., 2000; Nowak et al., 2009; Rinne et al., 2002). The highly selective H2 receptor antagonist famotidine, when co-administered with L-DOPA in MPTP-lesioned macaque monkeys, can reduce the severity of peak-dose L-DOPA-induced chorea and can prolong the anti-parkinsonian action of L-DOPA (Johnston et al., 2010). However, the mechanism for this effect and potential therapeutic limits are unknown, as H3 agonists that decrease choreic LID can also increase dystonic forms of LID (Gomez-Ramirez et al., 2006).

In light of our previous studies implicating striatal ChI pathology in LID, and considering reports of changes in histamine signaling associated with PD and LID, we data-mined the gene expression profile of striatal ChIs from the results of a study using translating ribosome affinity purification in BAC transgenic mice (Doyle et al., 2008) and found a relatively high and selective expression of H2 receptors. This result provided a strong rationale for targeting H2 receptors as a means to modulate ChI activity and reduce LID in patients with PD. To test this model, we used the selective H2 receptor antagonist, famotidine, to evaluate the contribution of these receptors to ChI excitability, and to examine the effects of H2 antagonism on behavioral measures of LID in two mouse models of PD.

## Materials and Methods

### Animals

All procedures were approved by the Institutional Animal Care and Use Committee of the University of Chicago. Animals were housed in a pathogen-free environment on a 12-hour light / dark cycle with unlimited access to food and tap water.

Two animal models of PD were used for these experiments. One was C57BL/6 mice unilaterally lesioned with 6-hydroxydopamine (6-OHDA), as described previously (Won et al., 2014). And the second was homozygous Pitx3*^ak/ak^* mice that lack nigrostriatal dopamine projections from birth, which we and others have also used previously (Ding et al., 2011, 2007; (Solis et al., 2014). The main differences between these models are the timing of the dopamine loss (from birth vs after lesion) and the pattern of denervation, with the Pitx3^ak/ak^ mice showing bilateral DA denervation predominantly in the dorsal striatum, while 6-OHDA lesioned mice generally show unilateral striatal DA denervation across the entire striatum. LID induction required chronic treatment with L-DOPA by daily injections, as outlined below. The L-DOPA dosing for each model was determined empirically as that necessary to induce dyskinetic behavior. Differences in dosing between these animal models were due to differential denervation patterns and possible developmental adaptations.

### Electrophysiology

Parasagittal brain slices (250 μm) were taken from adult mice pretreated with saline or L-DOPA as outlined in the behavioral testing sections below. Mice were anesthetized with isoflurane (Baxter) and then rapidly decapitated. Brains were removed and transferred into ice-cold low-sodium, sucrose-artificial cerebrospinal fluid (ACSF) containing (mM): 200 Sucrose, 25 NaHCO_3_, 20 glucose, 10 ascorbic acid, 2.5 KCl, 2.5 CaCl_2_ • 2H_2_O, 1 MgCl_2_ • 6H_2_O, 1 NaH_2_PO_4_, saturated with 95% CO_2_ / 5% O_2_, pH 7.3. Separate parasaggital slices containing dorsolateral striatum were taken from each hemisphere using a vibrating microtome (VT1000S, Leica Biosystems) and transferred into a 32° C circulating bath with ACSF (125 NaCl, 25 NaHCO_3_, 20 glucose, 2.5 KCl, 2.5 CaCl_2_ • 2H_2_O, 1 MgCl_2_ • 6H_2_O, 1 NaH_2_PO_4_,1 ascorbic acid, bubbled continuously with 95% CO2 / 5% O2, pH 7.3). During recording, slices were superfused with ACSF without ascorbate at a rate of 1.8 - 4 mL/min at room temperature. All recordings were performed between 40 min and 6 hrs following slicing.

Slices were transferred to a recording chamber with a volume of ∼1 mL and then visualized using an upright microscope (Axioskop, Carl Zeiss) with a water immersion 40× objective (Carl Zeiss) and a monochrome CCD camera (Hamamatsu). Recordings were performed using borosilicate glass pipettes (Warner Instruments) pulled on a P-97 Flaming/Brown micropipette puller (Sutter Instrument Company) with pipette resistance of 2 – 5 MΩ, and filled with filtered ACSF. Cell-attached current clamp recordings were performed on ChIs that were identified based on large cell diameter (>15 μm) and spontaneous action potential firing (0.1 – 7 Hz). Action potentials were measured using a Multiclamp 700A amplifier with a 10 kHz low-pass Bessel filter and a DigiData 1332A 16-bit data acquisition software sampling at 5 kHz and recorded using Clampex 9.2 software (Axon Instruments). Action potential data were analyzed off-line using MiniAnalysis (Synaptosoft). Spike amplitude thresholds were set to 20× baseline RMS noise levels, as determined by the analysis software. Integrated spike area and rise-time criteria were used eliminate random fluctuations that were independent of cell firing. Frequency of firing rate was determined in 10 second bins. Data were expressed as mean ± SEM.

Histamine (1 μM; Sigma-Aldrich) was bath applied for 5 min. In antagonist experiments, famotidine (1 μM; Envoy Therapeutics) dissolved in either DMSO or acidified deionized water was pre-applied for 5 min before histamine and antagonist co-application. The effect of histamine on firing rate was measured as the peak firing rate in a 1 min time window during time 1.5 – 8.5 min following onset of histamine bath application. Flow rate of drug administration was matched to the ACSF flow rate (1.8 - 4 mL/min).

### Behavior test on unilateral 6-OHDA lesioned mice

Wild type male, 7 month old C57BL/6 mice were unilaterally lesioned with 6-hydroxydopamine (6-OHDA) as described previously (Won et al., 2014). Briefly, mice were anesthetized and put into a stereotaxic device. Desipramine (25 mg/kg i.p., Sigma-Aldrich) was injected to protect norepinephrine neurons. Thirty min later, 2 μL of 2 mg/ml 6-OHDA (dissolved in 0.01% ascorbate in 0.9% saline, Sigma-Aldrich) was injected into left medial forebrain bundle (coordinates: anteroposterior, −1.3 mm; lateral, 1.3 mm; dorsoventral, −4.9 to −5.2 mm from the skull surface) via a 28-gauge stainless-steel cannula. Animals were allowed to recover for 4 weeks in their home cages before L-DOPA treatment.

Mice were then randomly divided into two groups, designated to receive either chronic L-DOPA or chronic vehicle treatment. The chronic L-DOPA group (n = 32) received L-DOPA methyl ester/benserazide injections (2/12.5 mg/kg dissolved in 0.9% saline, i.p.) either once a day or divided into two doses a day through the course the experiment. The chronic vehicle group (n = 15) received benserazide (12.5 mg/kg) only. Daily L-DOPA or vehicle injections continued for a total of 5 – 9 months. To confirm the induction of dyskinesia, chronic L-DOPA treated mice underwent behavioral testing following a test dose of 2 mg/kg L-DOPA on days 1, 8, 22 and 36 after the initial L-DOPA treatment. As in our previous studies, dyskinetic motor behavior was stronger at later time points (Ding et al., 2007). On behavioral testing days, animals received only the test dose of L-DOPA, but no maintenance injection. After day 36, the test dose was increased to 3 mg/kg L-DOPA and mouse behavior was videotaped for a span of 3 min every 20 min during a 2 hour period. L-DOPA-induced limb and axial dyskinesia was evaluated for 2 min at each 20 min interval by an observer blinded to experimental conditions using a previously described rating scale (Ding et al., 2011). Limb dyskinesia consists of abnormal, purposeless hyperkinetic movement of the contralateral forelimb while axial dyskinesia is characterized by a twisting of the neck and upper torso towards the side contralateral to the lesion. For experiments evaluating the effects of famotidine or dicyclomine on dyskinesia, the chronic L-DOPA treated group was divided into three groups which were balanced with respect to severity of dyskinesia. Group A received famotidine (Envoy Therapeutics, dissolved in sterile water acidified with 1 mM HCl and then adjusted to pH 6.5 with 1 mM NaOH, i.p.) at doses of 1, 3, and 10 mg/kg (increasing dosage every 2 days). Group B received dicyclomine (Sigma-Aldrich, dissolved in 0.9% saline, i.p.) at 45 mg/kg. Famotidine was administered 45 min, while dicyclomine was administered 30 min prior to L-DOPA test dose (See Figs 5A, 6A). Group C was given saline to serve as a vehicle control for famotidine or dicyclomine.

The stepping test was videotaped and scored to assess forelimb akinesia in chronic L-DOPA and chronic vehicle treated mice as described previously (Ding et al., 2011). The front paws of the mice were put on a treadmill as the belt moved forward for one full cycle while the torso and hindpaws were held up by the experimenter. Each mouse was tested in five nonconsecutive cycles. Stepping was scored by an observer blinded to treatment conditions. The number of left and right paw steps were counted for each of the 5 cycles and then averaged. In chronic L-DOPA treated mice, the stepping test was performed before and 1 hr following injection of 3 mg/kg L-DOPA. Famotidine (1, 3, or 10 mg/kg) or saline (vehicle) was administered 45 min prior to the test dose of 3 mg/kg L-DOPA.

To test the in vivo brain concentration and pharmacokinetic time course of famotidine in mice, we used MetaQuant microdialysis to measure striatal levels following systemic administration. We found a significant increase in the levels of famotidine in the striatum of non-lesioned mice after administration of 250 mg/kg i.p. The concentration of famotidine in the dialysate from the striatum peaked at 225 nM at 60-90 min. Given that the K_d_ of famotidine at the H2 receptor is 17 nM (Hill, 1990), we used a range of doses between 1 and 50 mg/kg to achieve maximal free striatal concentrations in the low nM range, where famotidine is highly selective for H2. Injections were timed such that the peak famotidine concentrations corresponded with the peak L-DOPA-induced dyskinetic behavior. As illustrated in Figs 5A and 6A, the L-DOPA injections occurred 45 min after famotidine administration. The doses used in the experiments did not produce sedation.

### Behavior Test on Homozygous *Pitx3^ak/ak^* Mice

Homozygous *Pitx3^ak/ak^* mice were obtained as described before (Ding et al., 2011, 2007). Homozygous *Pitx3^ak/ak^* mice have a mutation for retinal degradation (*Pde6brd1*), causing severely abnormal lens development resulting in blindness (van den Munckhof et al., 2003; Varnum and Stevens, 1968). They were identified by their small, closed eyes, even late in development.

*Pitx3^ak/ak^* mice (2-4 months of age) were randomly divided into two groups and matched with respect to age and gender. Group A received daily injections of L-DOPA/benserazide (25/12.5 mg/kg dissolved in 0.9% saline, i.p.) b.i.d. (n = 21). Group B received vehicle (benserazide 12.5 mg/kg, i.p.) only (n = 20). Exposure to L-DOPA lasted 5 – 9 months. To assess dyskinetic behavior, *Pitx3 ^ak/ak^* mice were placed in a clear upright Plexiglas cylinder. The number of rearing events and duration of abnormal paw movements were analyzed within a 2 min period 15 min following L-DOPA test dose (25 mg/kg). Abnormal paw movements that were scored consisted of front paw dyskinesia, three paw dyskinesia, and total dyskinesia, and each was analyzed separately. Behavioral testing was performed and videotaped on days 1, 8, 22, 36, and 40 to confirm the onset of paw dyskinesia as described before (Ding et al., 2011, 2007).

Group A *Pitx3^ak/ak^* mice were divided into three groups. The first group received saline (vehicle) 30 or 45 min prior to L-DOPA (25 mg/kg) test dose (n = 7). The second group received famotidine (2, 10, or 50 mg/kg, increasing in dosage every 2 days, i.p.) 45 min prior to L-DOPA (25 mg/kg) test dose. The third group received dicyclomine (15 or 45 mg/kg, increasing in dosage every 2 days, i.p.) 30 min prior to L-DOPA (25 mg/kg) test dose.

### Data Analysis

Data for behavioral and electrophysiological assessments are expressed as mean ± SEM. All firing rate data were normalized to the average firing rate across the six 10s bins prior to any exposure to drug. Electrophysiological analyses were performed using MiniAnalysis 6.0.3 (Synaptosoft). Statistical analyses were performed using SigmaStat, v.2.03 and graphed using SigmaPlot v.12.0. An unpaired t-test was performed to determine statistical differences between percent inhibition in electrophysiological measures, while one or two way ANOVA was used for behavioral measures.

## Results

### Striatal cholinergic interneurons express histamine receptors

**Figure 1:**
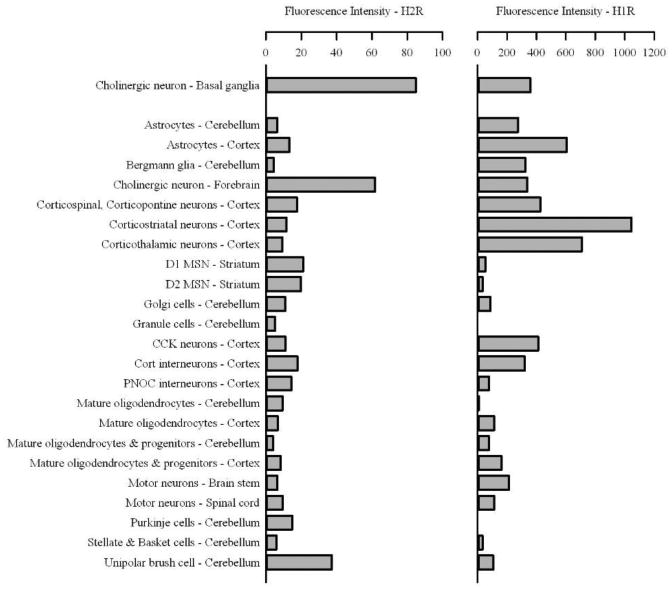
Expression of excitatory post-synaptic histamine receptors in striatal ChIs Striatal ChIs express both histamine H1 and histamine H2 receptors. Published BAC-TRAP microarray data (Doyle et al., 2008) was searched to determine which histamine receptor mRNAs are co-immunoprecipitated with eGFP-tagged ribosomes expressed under the control of the choline acetyltransferase promoter exclusively in ChIs within the striatum. The mean fluorescent intensities of Affymetrix probesets corresponding to H1 [Hrh1 (1438494_at)] and H2 [Hrh2 (1423639_at)] in ChIs were 359.5 and 85.0, indicating both mRNAs were co-purified with the ribosomes. Histamine H1 and H2 receptor mRNA levels were compared across multiple cell types. Histamine H1 (Hrh1) mRNA was recovered with tagged ribosomes in several cell types at levels the same or higher than in striatal ChIs. Histamine H2 (Hrh2) mRNA had the highest hybridization signal in striatal ChIs.

Drawing upon our previous results showing enhanced excitability of striatal ChIs during LID along with the therapeutic effects of famotidine in relieving LID symptoms, we mined the database from a study using translating ribosome affinity purification (TRAP) (Doyle et al., 2008), where the translated mRNA of genetically defined populations of neuronal and glial cells, including striatal ChIs, were characterized by microarray analysis. We compared the hybridization signals across the 24 different cell types from the probes that detect mRNAs encoding excitatory post-synaptic H1 and H2 histamine receptors. Both H1 and H2 receptor mRNAs were recovered with ribosomes purified from striatal ChIs (Doyle et al., 2008), indicating that these receptors are expressed on ChIs. These data are in agreement with previous observation that histamine-induced depolarization of ChIs could be blocked largely by H1 antagonists, but only minimally by an H2-selective antagonist (Bell et al., 2000). The mRNA expression data support the observation that H1 is highly expressed in ChIs. In contrast, the lower-abundance H2 mRNA shows the highest expression levels in ChIs compared to other cell types, suggesting a more selective target compared to the widely expressed H1 receptor (Fig 1). Therefore, H2 antagonists may selectively reduce the sensitivity of ChIs to histamine while minimizing undesirable effects on other histamine dependent functions, such as sleep regulation or feeding behavior (Nuutinen and Panula, 2010).

### Histamine increases excitability of dorsolateral striatal cholinergic interneurons

**Figure 2:**
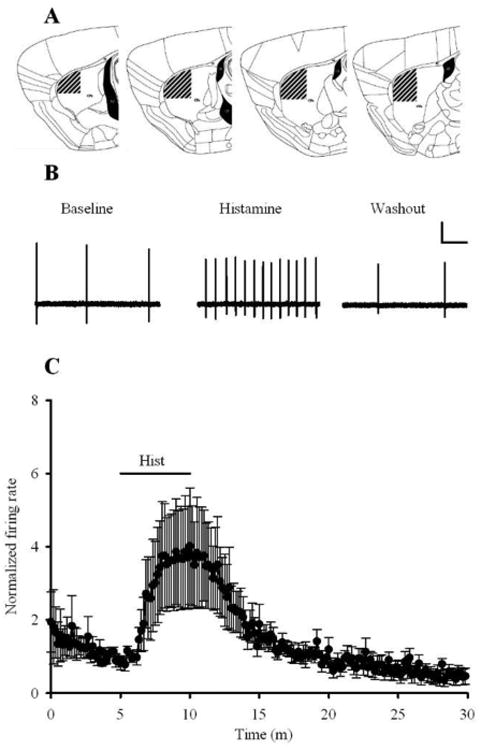
Histamine induced excitation in control dorsolateral striatal cholinergic interneurons (A) All cells analyzed for the following experiments were located in the dorsal, lateral, and rostral halves of the striatum, indicated by the striped area outlined above. Lateral half was defined by the coordinates 2.75 and 1.75 mm from midline. This diagram also represents the parasagittal slice orientation and approximate amount of brain tissue collected at the time of recording. (B) Representative cell-attached current clamp traces from ChIs located in the unlesioned hemisphere of 6-OHDA lesioned animals showing spontaneous action potential firing at baseline. Firing rate increases upon exposure to bath application of 1 μM histamine. Return to ACSF superfusion causes firing rate to return to baseline levels. Vertical scale bar: 1 mV. Horizontal scale bar: 1 s. (C) 1 μM histamine induces a reversible 4.11 ± 1.42 fold increase in action potential firing rate. Histamine induced increase is represented as fold change difference compared to the average firing rate in the min prior to bath application. (n = 6. Error bars represent ± SEM).

We assessed the effects of histamine on the excitability of rostral dorsolateral striatal ChIs in parasagittal slices taken from two different mouse models of PD: unilateral 6-OHDA lesion and *Pitx3^ak/ak^* mutation. All mice were subjected to daily injections of either L-DOPA or vehicle for 5-9 months. Cell-attached current clamp recordings were performed on ChIs that were identified based on cell morphology (cell diameter > 15 μm) and electrophysiological characteristics (spontaneous action potential firing rate between 0.1 and 7 Hz)(Kawaguchi, 1993). All ChIs were located in the rostral half of the dorsolateral striatum (Fig 2A). Our control recordings were conducted in the unlesioned hemisphere of 6-OHDA lesioned animals following chronic vehicle treatment. Bath application of histamine (1 μM) increased spontaneous action potential firing in these neurons (411% ± 142% of baseline, n = 6). This increase in excitation was reversed upon washout with ACSF (Fig. 2B, C). These data are consistent with previous reports demonstrating that bath application of histamine induces excitatory currents in whole cell voltage clamp recordings, and depolarization accompanied by action potential firing in current clamp recordings from previously silent rat striatal ChIs (Bell et al., 2000).

### Chronic L-DOPA treatment enhances histamine H2 effect on cholinergic interneuron excitability in 6-OHDA lesioned mice

**Figure 3:**
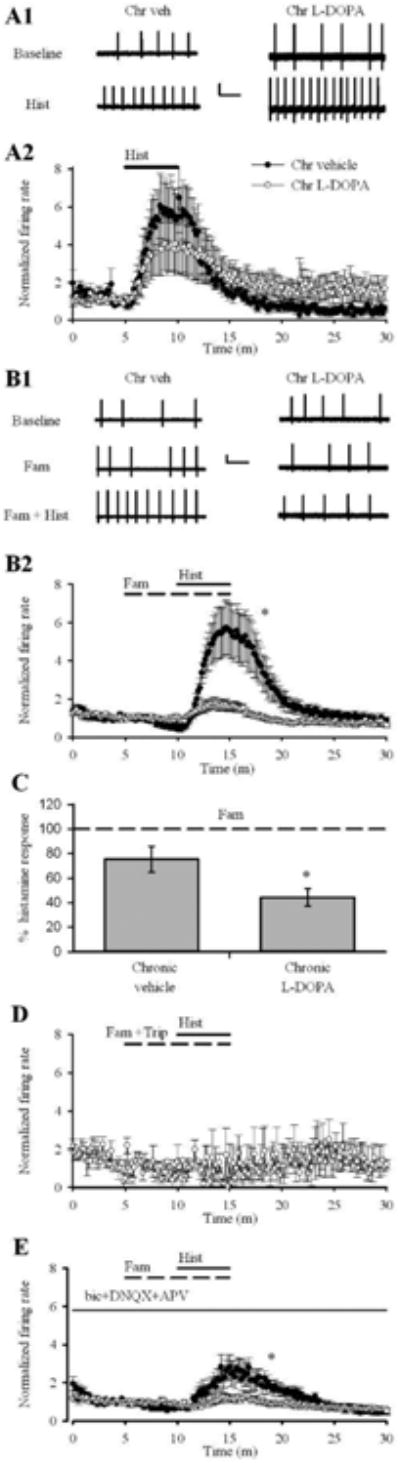
H2 antagonism is more effective at blocking the histamine induced increase in ChI firing in dyskinetic 6-OHDA lesioned mice (A) 1 μM histamine increases action potential firing rate among ChIs located in the lesioned hemisphere of 6-OHDA lesioned mice, both chronically treated with vehicle and with L-DOPA. A1 shows sample traces (Scale: 1 mV, 1 s for A1 and B1). A2 shows a histogram of average firing rates. (Vehicle: n = 6; L-DOPA: n = 8). (B) In chronic vehicle-treated animals, pre-application of 1μM famotidine had no effect on the histamine induced increase in firing rate. In dyskinetic L-DOPA treated animals however, 1μM famotidine inhibited the histamine induced increase in ChI firing. (Vehicle: n = 16; L-DOPA: n = 10. Data were analyzed using Student's t-test. * p < 0.05). (C) Summary data illustrating that pre-application of 1 μM famotidine inhibits the histamine induced increase in action potential firing in chronic L-DOPA treated animals but not vehicle-treated animals. Dotted line indicates peak histamine induced increase in firing rate, normalized for each treatment group. Bars represent percent change of the histamine response while in the presence of famotidine (* p < 0.05 between treatment groups. Error bars represent ± SEM). (D) Pre-application of both H1 and H2 receptor antagonists completely occlude the excitatory effects of histamine. (n = 3) (E) Famotidine blocked the histamine induced excitation in dyskinetic mice in the presence of synaptic excitatory (DNQX, APV) and inhibitory (GABA) antagonists (Vehicle: n = 7; L-DOPA: n = 5. * p < 0.05 between treatment groups).

Following unilateral 6-OHDA lesion, chronic L-DOPA treatment induces LID-like behaviors, and only animals that demonstrated LID behaviors were used for electrophysiology experiments (Ding et al., 2007). In the chronic vehicle treated group, only mice exhibiting a significant unilateral stepping deficit (indicative of dopamine deficiency) (Chang et al., 1999) were used for electrophysiology. We found a similar effect of histamine (1 μM) on the excitability of ChIs in brain slices from the lesioned hemisphere of either chronic vehicle treated mice (633% ± 167% of baseline, n = 6) or dyskinetic chronic L-DOPA treated animals (415% ± 147% of baseline, n = 8; Fig 3A. Data were analyzed using Student's t-test. t(12) = 0.95, p > 0.05).

To evaluate the contribution of H2 receptors to the excitatory effects of histamine, slices were pretreated with famotidine (1 μM) for 5 min before histamine (1 μM) and famotidine co-application. Famotidine alone did not produce a significant difference in the firing rate compared to baseline. In slices from animals that were chronically treated with L-DOPA the co-application of famotidine and histamine resulted in less histamine mediated excitation than histamine alone, but not in slices from the chronic vehicle treated group (Fig 3B; t(24) = 2.24, p < 0.05). In vehicle treated animals, famotidine showed a relatively weak inhibitory effect (75.3% ± 10.7% of histamine response), while ChIs in slices from dyskinetic animals displayed significant famotidine inhibition (44.3% ± 7.1% of histamine response; Fig 3C; t(23) = 2.16, p < 0.05). Together, these data indicate that H2 receptors contribute more strongly to ChI neuron excitation in mice expressing LID compared to chronic vehicle treated animals.

The histamine mediated excitation observed in the presence of famotidine can be attributed to activation of H1 receptors. This is supported by recordings where co-application of famotidine with the H1R antagonist triprolidine completely inhibited histamine induced excitation (Fig 3D). The famotidine alone effects suggest that H1 receptors are predominantly responsible for the excitatory effects of histamine on ChIs from vehicle treated animals (Fig 1). It is notable that H1 receptors are also activated at lower histamine concentrations (Munakata and Akaike, 1994). In tissue slices from chronic L-DOPA-treated animals, both H1 and H2 receptors contribute to histamine-induced excitation, but the relative contribution of H2 receptors to this phenomenon is significantly enhanced relative to ChIs from vehicle treated animals. Co-application of famotidine with the H1R antagonist triprolidine significantly blocks histamine induced excitation (145% ± 40% of baseline; Fig 3D, n = 3). As a result, we conclude that histamine increases cell firing by activation of both H1 and H2 receptors.

To assess the possible contribution of glutamatergic and/or GABA-ergic transmission to histamine excitation of ChIs, recordings were conducted in the presence of DNQX (10 μM) and APV (50 μM) to block AMPA and NMDA glutamate receptors, respectively, as well as bicuculline (20 μM) to block GABA_A_ receptors. Under these conditions, we observed an enhanced H2 mediated component of histamine excitation in the dyskinetic chronic L-DOPA treated animals (Fig 3E; t(10) = 1.328, p < 0.05), indicating that the LID-related H2 excitation of these neurons is mediated by direct activation of ChIs rather than by indirect presynaptic effects. The magnitude of the histamine excitation is lower than that seen without blockers of glutamate and GABA transmission. This could be due to loss of tonic glutamate excitation reported previously (Feng et al., 2014), or a decrease in the impact of excitatory inputs from other ChIs, as the glutamate co-released at those inputs would not contribute to increased firing rates (Gras et al., 2008; Nelson et al., 2014).

### Chronic L-DOPA treatment enhances histamine H2 effect on cholinergic interneuron excitability in *Pitx3^ak/ak^* mice

**Figure 4:**
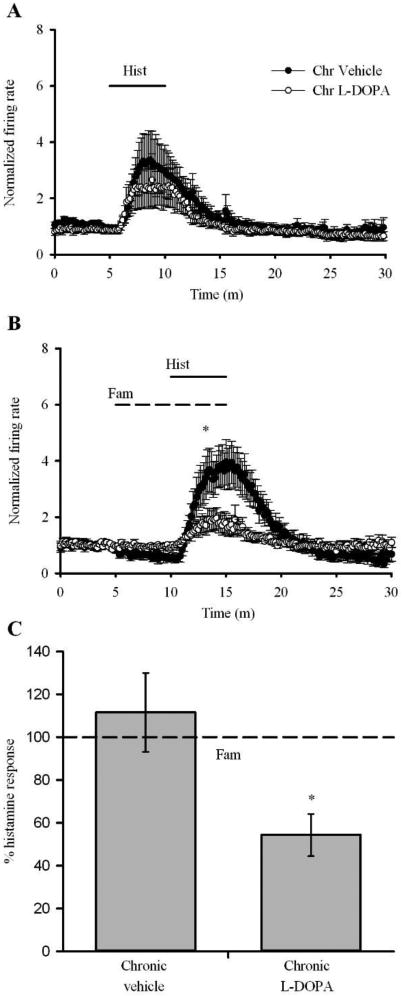
H2 antagonism is more effective at blocking the histamine induced increase in ChI firing in dyskinetic *Pitx3^ak/ak^* mice (A) 1 μM histamine increases action potential firing rate among ChIs located in both hemispheres of *Pitx3^ak/ak^* mice, both chronically treated with vehicle and with L-DOPA. (Vehicle: n = 8; L-DOPA: n = 9). (B) In nondyskinetic, chronic vehicle-treated animals, pre-application of 1μM famotidine had no effect on the histamine induced increase in firing rate. In dyskinetic L-DOPA treated animals however, 1μM famotidine inhibited the histamine induced increase in ChI firing. (Vehicle: n = 10; L-DOPA: n = 8. Data were analyzed using a two-tailed Student's t-test. * p < 0.05). (C) Summary data illustrating that pre-application of 1 μM famotidine inhibits the histamine induced increase in action potential firing in chronic L-DOPA treated dyskinetic animals but not vehicle-treated animals. Dotted line indicates peak histamine induced increase in firing rate, normalized for each treatment group. Bars represent percent change of the histamine response while in the presence of famotidine. (* p < 0.05 between two treatment groups. Error bars represent ± SEM).

To further examine the role of H2 receptors in LID, we repeated the electrophysiological tests using *Pitx3^ak/ak^* (aphakia) mice. As with the 6-OHDA lesioned mice, the *Pitx3^ak/ak^* mice were chronically treated with vehicle or L-DOPA (see Methods). In dorsolateral ChIs from both striatal hemispheres of *Pitx3^ak/ak^* mice, histamine alone induced an increase in tonic firing in both chronic vehicle treated (376% ± 101% of baseline, n = 8) and dyskinetic chronic L-DOPA treated animals (356% ± 108% of baseline, n = 9; Fig 4A; t(15) = -0.16, p > 0.05).

Famotidine application alone did not significantly alter the firing rate compared to baseline. As seen in 6-OHDA lesioned animals, co-application of famotidine with histamine revealed a differential degree of histamine mediated excitation depending on treatment group (Fig 4B; t(16) = 2.3, p < 0.05). The inhibition of the histamine response by famotidine was stronger in tissue from *Pitx3^ak/ak^* mice expressing LID (54.3% ± 9.8%) compared to slices from chronic vehicle treated animals (111.5% ± 18.4%; Fig 4C; t(16) = 2.54, p < 0.05). Together, the data from both PD model animals indicate that the H2 receptor contribution to histamine mediated excitation of ChIs is stronger in chronic L-DOPA-treated relative to vehicle-treated mice.

### Famotidine or dicyclomine decreases behavioral expression of LID in 6-OHDA lesioned mice

**Figure 5:**
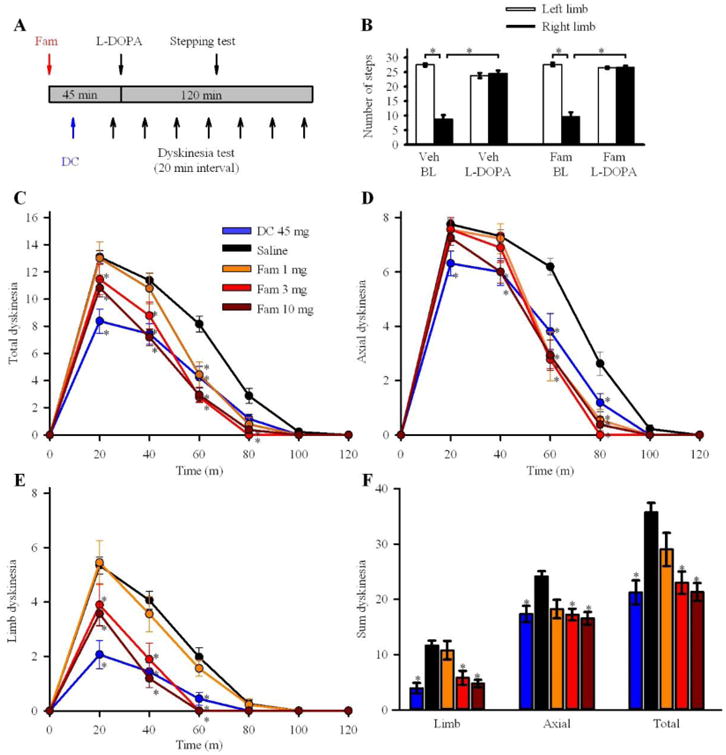
Famotidine decreases L-DOPA-induced dyskinesia in 6-OHDA lesioned mice (A) Timeline for the drug treatment and behavioral tests on 6-OHDA lesioned mice. Chronic L-DOPA treated 6-OHDA lesioned mice were pretreated with famotidine 45 min or dicyclomine 30 min prior to L-DOPA. Control groups were injected with saline with the same interval as famotidine or dicyclomine prior to L-DOPA. Behavioral tests were performed 60 min after L-DOPA injection for stepping test, and every 20 min for a total of 120 min for assessment of dyskinesia. (B) Stepping test for 6-OHDA mice pretreated with vehicle or famotidine (10 mg/kg) 45 min prior to L-DOPA (3 mg/kg). L-DOPA improved unilateral akinesia regardless of pretreatment. (n = 10 animals in each group. Data were analyzed with a one-way ANOVA on ranks. * p < 0.05). Famotidine or dicyclomine decreased the L-DOPA-induced total dyskinesia (C), axial dyskinesia (D), and limb dyskinesia (E) in 6-OHDA lesioned mice chronically treated with L-DOPA. (F) Summary of the effect of famotidine or dicyclomine on L-DOPA-induced dyskinesia in the 6-OHDA lesioned mice. (DC – dicyclomine. DC: n = 16, Saline: n = 32, Fam 1mg/kg: n = 9, 3 mg/kg: n = 9, 10 mg/kg: n = 16. Data were analyzed with a two-way ANOVA. Data from panel F was analyzed with Kruskal-Wallis ANOVA on Ranks. * p < 0.05 compared with saline treated group. Error bars represent ± SEM).

We then tested whether famotidine could reduce the LID behaviors associated with chronic L-DOPA treatment (see Methods). Mice received an i.p. injection of famotidine 45 min prior to L-DOPA test dose. The peak in striatal famotidine level occurs 60-90 min after injection. The injection time course was designed such that the peak famotidine level correlates with the peak L-DOPA-induced dyskinetic behavior (Fig 5A). Famotidine injections at the highest dose tested (10 mg/kg) did not alter the L-DOPA-induced improvement of locomotion, or relief of akinesia in the 6-OHDA lesioned mice, as measured in a forelimb stepping test (Fig 5B; n = 10 in each group. Data were analyzed using a one-way ANOVA on ranks. F(2, 38) = 71.012, p < 0.05).

A test dose dose of 3 mg/kg L-DOPA was given to all mice prior to LID testing. Assessment of ‘total dyskinesia’ included measurement of both axial dyskinesia (twisting of the neck and upper body toward the side contralateral to the lesion) and limb dyskinesia (purposeless hyperkinetic movement of the contralateral forelimb). The effects of famotidine on LID were compared with that of dicyclomine, a muscarinic antagonist that is known to attenuate LID associated behaviors (Ding et al., 2011).

Mice receiving famotidine exhibited significantly less severe L-DOPA-induced total dyskinesia. The higher doses (3 and 10 mg/kg) showed a decrease at 20, 40, 60 and 80 min following L-DOPA injection, while 1 mg/kg showed a decrease only at the 60 min time point (Fig 5C; Saline: n = 32, famotidine 1 mg/kg: n = 9, 3 mg/kg: n = 9, 10 mg/kg: n = 16; dicyclomine 45 mg/kg: n = 16. Data were analyzed using a two-way ANOVA. F(3, 24) = 5.428, p < 0.05). These reductions were similar to those seen following dicyclomine treatment (45 mg/kg) (Fig 5C). Summing dyskinesia scores over the entire time frame of LID testing from 0 to 120 min revealed that the higher doses of famotidine (3 and 10 mg/kg) or dicyclomine (45 mg/kg) significantly reduced total dyskinetic behavior (Fig 5F).

A significant decrease in axial dyskinesia was observed following pretreatment. The high dose famotidine (10 mg/kg) showed a decrease at 40, 60, and 80 min, while 1 and 3 mg/kg showed a decrease at 60 min and 80 min (Fig 5D; F(3, 24) = 4.755, p < 0.05). Sum of axial dyskinesia over the entire time course was decreased following pretreatment with famotidine (3 and 10 mg/kg, Fig 5F). Dicyclomine (45 mg/kg) significantly decreased the L-DOPA-induced axial dyskinesia at 20, 40, and 60 min, as well as the sum of axial scores across the time course of LID testing (Fig 5D, F).

Limb dyskinesia is decreased in mice receiving famotidine (3 mg/kg, 10 mg/kg) at 20, 40, and 60 min (Fig 5E; F(3, 24) = 6.373, p < 0.05) compared to mice receiving saline. Sum of the limb dyskinesia across the time course was also decreased following famotidine treatment (Fig 5F). The antidyskinetic effect of high dose famotidine (10 mg/kg) was comparable to the dicyclomine (45 mg/kg) mediated decrease in dyskinesia.

Together these data show that famotidine treatment reduced peak LID expression and its duration, with the strongest effects seen at the higher doses. The greatest fractional effects of the highest famotidine doses were seen in limb dyskinesia, with greater than 50% reduction in the sum L-DOPA effect. Axial dyskinesia behaviors were more pronounced, and were less sensitive to the famotidine treatment - however, the famotidine induced improvement of LID was similar to that seen with dicyclomine treatment.

### Famotidine or dicyclomine decreases behavioral expression of LID in *Pitx3^ak/ak^* mice

**Figure 6:**
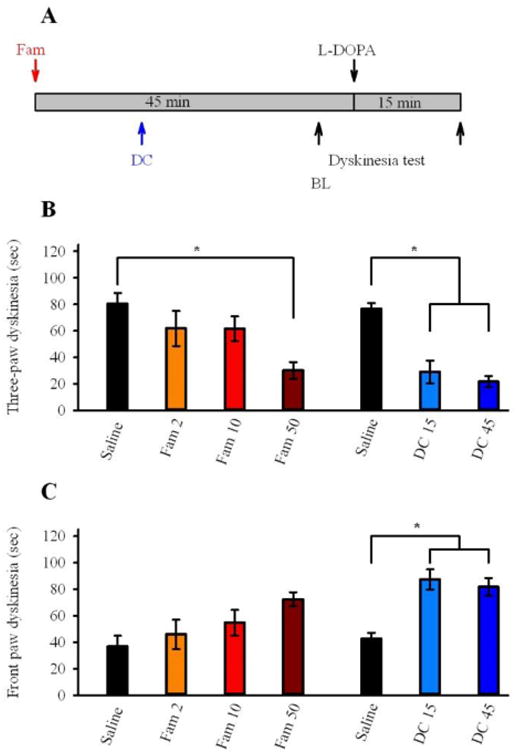
Famotidine decreases L-DOPA-induced dyskinesia in *Pitx3^ak/ak^* mice (A) Timeline for the drug treatment and behavioral tests on *Pitx3^ak/ak^* mice. Chronic L-DOPA (25 mg/kg) treated *Pitx3^ak/ak^* mice were pretreated with famotidine 45 min or dicyclomine 30 min prior to L-DOPA. Control groups were injected with saline with the same interval as famotidine or dicyclomine prior to L-DOPA. Behavioral tests were performed 15 min after L-DOPA injection to quantify duration of three-paw dyskinesia (B) and front paw dyskinesia (C) (DC- dicyclomine. n = 7 animals in each group. Data were analyzed with a one-way ANOVA. * p < 0.05 compared with saline treated group. Error bars represent ± SEM).

Behavioral testing was also carried out on chronic L-DOPA treated *Pitx3^ak/ak^* mice that expressed dyskinesia. Animals were pretreated with famotidine, dicyclomine or saline prior to L-DOPA test dose (25 mg/kg) and behavioral testing (Fig 6A; n = 7 in each group). Two types of abnormal paw movements were analyzed: three-paw dyskinesia and front paw dyskinesia. Since three-paw dyskinesia is observed after higher doses of L-DOPA and with longer exposure to L-DOPA, we believe it represents a more severe form of dyskinesia (Ding et al., 2007). Famotidine (50 mg/kg) significantly reduced three-paw dyskinesia, an effect mimicked by dicyclomine (Fig 6B; Data were analyzed using a one-way ANOVA. Famotidine, F(3, 12) = 3.478, p < 0.05. Dicyclomine, F(3, 12) = 17.633, p < 0.05). As the three-paw form of dyskinesia diminished, the animals displayed a trend towards an increase in front paw dyskinesia, which was also seen with dicyclomine (Fig. 6C; Famotidine, F(3, 12) = 2.061. Dicyclomine, F(3, 12) = 10.797. p < 0.05). Taken together, the pattern of dyskinesia in chronic L-DOPA treated *Pitx3^ak/ak^* mice shifts from three-paw to front paw in response to treatment with famotidine or dicyclomine, indicating that both drugs decrease the severity of dyskinesia.

## Discussion

Our results support the idea that H2 antagonists can selectively modulate the hyperactive ChI and may decrease the expression of dyskinetic behavior following chronic L-DOPA treatment. These studies were motivated by results of mining BAC-TRAP gene expression data, which suggested that ChIs express histamine H2 receptor mRNA at higher levels than other cell types. Using two PD mouse models, 6-OHDA lesion and *Pitx3^ak/ak^*, we found that histamine induced a strong increase in firing of ChIs in the dorsolateral striatum. After chronic L-DOPA treatment to induce LID, we found that H2 receptors contributed much more profoundly to histamine-induced excitation relative to vehicle-treated controls. Consistent with these data, we found that systemic administration of the H2 antagonist famotidine decreased the behavioral expression of dyskinesia following a test dose dose of L-DOPA.

These data suggest that changes in H2 receptor signaling in dorsolateral striatal ChIs contribute to LID. We previously demonstrated that these neurons display a stronger excitatory response to dopamine in LID (Ding et al., 2011). Here, we extend those findings to show that H2 histamine receptor effects on ChIs are enhanced in animals that display LID after chronic L-DOPA treatment. We utilized two different complementary models of mouse dyskinesia to demonstrate the effects of H2 antagonist. The H2 antagonist famotidine decreased LID dramatically in a 6-OHDA unilateral lesion model of PD. In *Pitx3^ak/ak^* mice, a high dose of famotidine was found to shift the behavior from three-paw dyskinesia to the less severe front paw dyskinesia. Since front paw dyskinesia develops early in L-DOPA treatment and at lower doses compared to three-paw dyskinesia, these data show that famotidine decreases the severity of LID in *Pitx3^ak/ak^* mice. Additionally, the *Pitx3^ak/ak^* mouse model exhibits less striatal dopamine depletion compared to the 6-OHDA lesion (Conti et al., 2014; Hwang et al., 2003). *Pitx3^ak/ak^* mice require a higher dose of L-DOPA to induce behavioral response (Espadas et al., 2012; Hwang et al., 2005), and it follows that the L-DOPA induced changes in H2 responses are not the same as those induced in lesioned animals. As a result, a higher dose of famotidine was necessary to decrease LID in *Pitx3^ak/ak^* mice. The data from both models support the idea that H2 antagonists may be a therapeutic adjunct in alleviating the symptoms of LID.

Acetylcholine activates both nicotinic and muscarinic receptors, and blocking either of these receptor classes reduces LID. Dyskinetic 6-OHDA lesioned rats chronically treated with the non-selective neuronal nicotinic receptor antagonist mecamylamine display a dramatic reduction in abnormal involuntary movements (Bordia et al., 2010). Chronic nicotine administration also decreases abnormal involuntary movements, which is likely due to receptor desensitization (Bordia et al., 2008). It is unclear where the nicotinic receptors that contribute to dyskinesia are located within the motor pathway. Additionally, the anti-muscarinic compound, dicyclomine, alleviates severity of dyskinesia in chronic L-DOPA treated *Pitx3^ak/ak^* mice and in 6-OHDA lesioned mice (Ding et al., 2011). In a small cohort of humans, co-administration of L-DOPA with the anticholinergic etybenzatropine decreased the severity of end-of-dose dyskinesia, and also increased the duration of action of L-DOPA (Pourcher et al., 1989) although older studies have noted worsening of LID by anticholinergics (Birket-Smith, 1974). Collectively, these results lend support to the notion that a hypercholinergic striatum may contribute to the expression of LID.

Histaminergic neurons in the tuberomamillary nucleus (TMN) are tonically active cells that send projections to the striatum (Haas et al., 2008; Köhler et al., 1985). Dopamine directly excites TMN neurons via both the dopamine D1 and D2 receptors (Yanovsky et al., 2011), which likely increases histamine release in the striatum. Histamine then activates excitatory H1 and H2 receptors on ChIs to increase cell excitability, and consequently, acetylcholine release in the striatum. H1 receptors couple to the G-protein G_q/11_ to activate PLC, whereas H2 receptors stimulate cAMP production by G_s_ activation of adenylyl cyclase (Haas et al., 2008). Ultimately, activation of either H1 or H2 receptors increase striatal ChI excitability by closure of K^+^ channels (Munakata and Akaike, 1994). This explains how L-DOPA, following enzymatic conversion into dopamine, increases striatal cholinergic tone via histaminergic signaling.

Compared to the H2 receptor, the H1 receptor is expressed at much higher levels (Fig 1), and also exhibits a higher affinity for histamine. Therefore, we expect that in the naïve animal, histamine induced excitation of ChIs will occur predominantly via activation of H1 receptors with minimal contribution from H2. In line with this prediction, Bell and coworkers observe only slight depolarization in naive rats following H2 activation, but robust action potential firing with H1 activation (Bell et al., 2000). However, dyskinesia from chronic L-DOPA treatment may induce a shift in the signaling pathway away from H1 and towards H2. This accounts for the robust H1-mediated excitation in vehicle-treated mice, but a strong contribution of H2 in dyskinetic mice (Figs 3 and 4). This is a functional effect that may not necessarily accompany changes in H2 receptor numbers (Martinez-Mir et al., 1993).

Histamine modulates excitability of striatal medium spiny neurons, primarily through H3 receptor-mediated suppression of presynaptic glutamate and GABA inputs (Prast et al., 1999)(Ellender et al., 2011). In contrast, histamine excitation of striatal ChIs appears to be predominantly through activation of somatic H1 and H2 receptors, as shown by the blockade of histamine excitation with H1 and H2 inhibitors (Fig 3D). In addition, the presynaptic H3 receptors are generally inhibitory and suppress neurotransmitter release. In the slice preparation, inhibitors of either GABA_A_ or glutamate receptors have no effect on the firing rate of ChIs (Bennett and Wilson, 1999). In our recordings, ChI firing rate was unaffected by bath application of bicuculline (10 μM; data not shown). Thus, we conclude that histamine excitation of ChIs occurs through direct activation of these neurons, and that the LID-associated increases in the H2 response occurs specifically on these neurons.

Our study is a powerful example of the utility of data mining gene expression studies to identify neuron-specific expression patterns for therapeutic discovery. Our results suggest that H2 antagonists may provide a novel tool to modulate the hyperactivity of striatal cholinergic neurons. A better understanding of the contribution of cholinergic and histaminergic signaling to striatal physiology, particularly in the context of LID and more selective and potent H2 antagonists may lead to new, more effective pharmacotherapies for basal ganglia disorders involving hyperactive ChIs.

## Conclusions

Although L-DOPA is the most effective therapy to alleviate PD, chronic treatment often leads to the development of debilitating dyskinesia. This dyskinesia is associated with excessive cholinergic signaling within the striatum. Here, we report an increase in the H2 receptor component of histamine mediated excitation of ChIs associated with LID. The behavioral expression of dyskinesia is decreased in the presence of an H2 receptor antagonist. These results were observed in two different mouse models of LID. Collectively, these findings indicate that H2 antagonists may be a useful therapeutic tool to modulate ChI activity, which may have implications for conditions such as LID in PD and dystonia.

## Highlights

- BAC-TRAP data indicate relatively selective expression of H2R on ChIs.
- Histamine H2R responses are elevated in ChIs in mice expressing LID.
- Histamine H2R antagonists decrease electrophisological activity of ChI and behavioral expression of LID.
- H2R effects were observed in two different mouse models of LID.
- We report a novel approach based on gene expression data to modulate specific cell type in the CNS, which is applicable for basal ganglia disorders such as PD and dystonia.

## Abbreviations

6-OHDA: 6-hydroxydopamine
ChI: cholinergic interneuron
LID: L-DOPA-induced dyskinesia
PD: Parkinson's disease
TMN: tuberomamillary nucleus
TRAP: translating ribosome affinity purification
